# Impact of Motion-Dependent Errors on the Accuracy of an Unaided Strapdown Inertial Navigation System

**DOI:** 10.3390/s23073528

**Published:** 2023-03-28

**Authors:** Krystian Borodacz, Cezary Szczepański

**Affiliations:** Łukasiewicz Research Network—Institute of Aviation, al. Krakowska 110/114, 02-256 Warszawa, Poland

**Keywords:** INS, IMU, inertial, navigation, accuracy, sensors, model, error budget, analysis, carouseling effect

## Abstract

The selection of an appropriate measurement system for an inertial navigation system requires an analysis of the impact of sensor errors on the position and orientation determination accuracy to ensure that the selected solution is cost-effective and complies with the requirements. In the current literature, this problem is solved based on the navigation duration only by considering the time-dependent errors due to sensor bias and random walk parameters or by conducting numerous simulations. In the former case, oversimplifying the analysis will not allow accurate values to be determined, while the latter method does not provide direct insight into the emerging dependencies. In contrast, this article introduces an analytic approach with a detailed model. This article presents general formulas, also written in detail for the measurement system model adopted and various manoeuvres. Although general equations are complicated, the use of piecewise constant motion variables allow us to discern fragments of equations corresponding to individual error sources. The results confirm the effect the carouseling has on the reduction of navigation errors. The general formulas presented extend the potential to analyse the influence of the entire host vehicle motion, while the detailed formulas make dependencies between motion and navigational errors evident.

## 1. Introduction

Inertial navigation systems provide continuous localisation, even during an outage or failure of other navigational means. However, the influence of measurement system errors results in a position determination error that increases with time and distance travelled. On the other hand, as the accuracy of sensors increases, their price also increases rapidly. Thus, it is necessary to select an appropriate measurement system that provides a cost-effective solution and, at the same time, sufficient accuracy, as it may determine the success of the mission. For this purpose, it is necessary to thoroughly analyse how the measurement errors of the considered inertial measurement unit will influence the accuracy of the position determination.

As a first step, an estimate of the required sensors’ accuracy can be determined based solely on navigation duration by considering the time-dependent errors due to sensor bias and random walk parameters [1]. A more detailed analysis, however, requires consideration of errors that build up during motion due to the predicted trajectory and sensors’ sensitivity to motion parameters. However, to the best of the authors’ knowledge and according to the literature review, there is a deficiency in a detailed analysis of this problem in the literature.

In [2], it is shown that the most significant influence on navigation error is due to the measurement errors of the gyroscope. However, the analysis applies only to the stationary conditions when the spatial (non-gravitational) accelerations of inertial measurement unit (IMU) are much smaller than gravity acceleration. The analysis presented in [3] applies to the case of a moving base but considers only the orientation error. The models used are derived and presented analytically, highlighting the relationships involved. Equations include only a simplified gyroscope model, and neither article considers motion-dependent errors. Nevertheless, the influence of the motion turns out to be significant, and the effects are intriguing.

Article [4] presents the results of an experimental study of a rotating inertial navigation system, showing that the introduction of system rotation can significantly reduce navigation errors. Indeed, the method of reducing navigational errors by setting the system into continuous rotation is generally known as ‘carouseling’ [5]. In contrast, ref. [6] points out that in a practical implementation of a rotating system, additional orientation determination errors may arise.

The analysis of the navigational systems’ accuracy through the determination of the error equations is performed in [7], where the reduction of position determination errors as a result of navigation system rotation is presented. However, considerations are limited to the biases of accelerometers and gyroscopes. The effect of carouseling on gyroscope error propagation is also discussed in [8]. Although the presented analysis was only concerned with orientation error, in addition to bias, it includes an accurate random error model for gyroscopes. However, the gyroscope sensitivity and alignment errors are not considered. A more complex model of the measurement system is presented by [9], although the model is used to calibrate the measurement system rather than to analyse the accuracy of the navigation system.

The only article found that deals with navigation error analysis and considers motion-dependent errors is [10]. The models presented include an accurate IMU model, but the examination is conducted in simulation, so the dependencies involved are not as well highlighted. The model presented in [10] fits into one of the approaches described in [11], where equivalence between error propagation models derived for different navigation system mechanisation is presented.

To summarise the results found in the literature, the standard approach in analysing navigation accuracy is to consider only time-dependent errors and ignore the influence of other sources. On the other hand, articles that analyse the impact of motion either focus on the accuracy of determining orientation only or analyse navigation accuracy assuming simplified sensor models. However, simulation analysis of navigation errors for a complex IMU model does not allow direct insight and understanding of the dependencies involved.

This paper presents the results of orientation and position determination errors calculated for an inertial navigation system. Calculations are carried out for selected, different manoeuvres and the assumed model of the measuring system. This paper uses an approach similar to that presented in [10], but the relationships are analytically expanded to make the impact of motion-dependent errors evident. The emerging relationships are analysed and discussed. In the next section, the navigation and measurement system models used for the analysis are described, and navigation error propagation formulas are presented. Section 2 also describes the methodology used and the manoeuvre cases considered. Detailed parameters of the measurement system are also presented. In Section 3, the obtained results are presented and briefly discussed. A detailed discussion of the results is provided in Section 4. The final section contains conclusions.

## 2. Materials and Methods

Here, we present the considered inertial navigation system and the IMU modelling method adopted. We detail the error propagation model and the approach undertaken for its solution and analysis. The section concludes with a presentation of the assumptions made and comments on the methodology undertaken.

### 2.1. General Models

**Figure 1:**
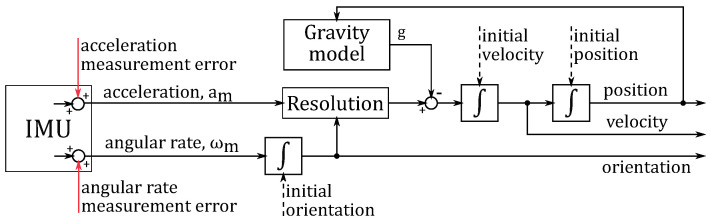
Block diagram of basic strapdown inertial navigation system (INS).

We analyse accuracy using a basic unaided strapdown inertial navigation system (INS), presented in Figure 1, with the assumption that the navigation coordinate system is inertial and orthogonal. The measurement system is an inertial measurement unit (IMU) consisting of a triad of orthogonal gyroscopes and a triad of orthogonal accelerometers. Their outputs are measured values of specific force and body angular rate expressed in the body coordinate system.

The values measured by the sensors include a measurement error described by an error model. The adopted model, presented in Equations (1) and (2), was developed based on information contained in standards [12,13,14] and the literature [15,16], wherein the effect of environmental conditions was neglected. In all of the following equations, vectors are written in bold, while matrices are in capital letters.

(1) $$\begin{array}{cc} \omega_{e}\left(t\right) & =\left(S_{\omega }\;M_{\omega }-I\right)\;\omega \left(t\right)+\omega_{b}+\Omega_{g}\;M_{\omega }\;f_{b}\left(t\right)+n_{\omega }, \end{array}$$

(2) $$\begin{array}{cc} a_{e}\left(t\right) & =\left(S_{a}\;M_{a}-I\right)\;f_{b}\left(t\right)+a_{b}+n_{a}, \end{array}$$

where

$\omega_{b}$, $a_{b}$ is the vector of measurement bias for gyroscopes and accelerometers;

$S_{\omega }$, $S_{a}$ is the matrix of sensitivity for gyroscopes and accelerometers;

$M_{\omega }$, $M_{a}$ is the matrix of input axes misalignment for gyroscopes and accelerometers;

$\Omega_{g}$ is the matrix of gyroscope sensitivity to acceleration; and

$n_{\omega }$, $n_{a}$ is the gyroscope and accelerometer measurement noise. 

**Figure 2:**
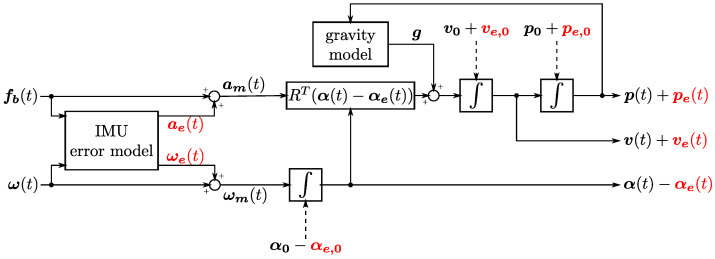
Block diagram of basic strapdown INS signals propagation for accuracy analysis.

In addition to measurement errors, the navigation system also suffers from errors in determining initial conditions and in determining navigation parameters. However, accurate knowledge of the local gravity vector is assumed. A block diagram of the navigation system under consideration with the symbols used is shown in Figure 2.

The following symbols are presented in Figure 2:

p(t), $p_{e}\left(t\right)$—position in the navigation coordinate system and the error of its determination;

v(t), $v_{e}\left(t\right)$—velocity in the navigation coordinate system and the error of its determination;

α(t), $\alpha_{e}\left(t\right)$—vector of rotation from the navigation coordinate system to the body coordinate system and the error of its determination;

$R\left(\alpha \left(t\right)\right)=\exp\left({\left[\alpha (t)\right]}_{\times }\right)$—matrix of rotation from the navigation coordinate system to the body coordinate system;

${\left[\alpha (t)\right]}_{\times }$—vector of rotation skew-symmetric matrix;

$f_{b}\left(t\right)=a\left(t\right)+\omega \left(t\right)\times u\left(t\right)-R\left(\alpha \left(t\right)\right)g$—specific force;

u(t)—velocity in the body coordinate system;

$a\left(t\right)=\dot{u}\left(t\right)$—body acceleration, expressed in the body coordinate system;

ω(t)—body angular rate expressed in the body coordinate system;

g—vector of gravity;

$\omega_{e}\left(t\right)$, $a_{e}\left(t\right)$—vectors of angular rate and acceleration measurement error. 

Subscript 0 within symbols used in Figure 2 denote initial values.

#### Error Propagation Model

The considered navigation system shown in Figure 2 is based on the following assumptions:

1. The navigation system is orthogonal and inertial;
2. Orientation errors during navigation are small;
3. Error products are neglected.

For such a system, the following error propagation model can be determined [16]:

(3) $$\begin{array}{cc} \;{\dot{\alpha }}_{e}\left(t\right) & =R^{T}\left(\alpha \left(t\right)\right)\;\omega_{e}\left(t\right), \end{array}$$

(4) $$\begin{array}{cc} {\dot{v}}_{e}\left(t\right) & =\alpha_{e}\left(t\right)\times f_{n}\left(t\right)+R^{T}\left(\alpha \left(t\right)\right)\;a_{e}\left(t\right), \end{array}$$

(5) $$\begin{array}{cc} \;\dot{p_{e}}\left(t\right) & =v_{e}\left(t\right). \end{array}$$

From the above-described navigation system and error propagation models, the general formulas for the orientation and position determination error were derived and are presented in Equations (6) and (7):

(6) $$\alpha_{e}\left(t\right)=\alpha_{e,0}+\int_{0}^{t}R^{T}\left(\alpha \left(t\right)\right)\;\left(\omega_{b}+\left(S_{\omega }\;M_{\omega }-I\right)\;\omega \left(t\right)\right)dt+\int_{0}^{t}R^{T}\left(\alpha \left(t\right)\right)\;\Omega_{g}\;M_{\omega }\;f_{b}\left(t\right)\;dt$$

(7) $$p_{e}\left(t\right)=p_{0,e}+t\;v_{0,e}+\int_{0}^{t}\int_{0}^{t}\alpha_{e}\left(t\right)\times R^{T}\left(\alpha \left(t\right)\right)\;f_{b}\left(t\right)\;dt\;dt+\int_{0}^{t}\int_{0}^{t}R^{T}\left(\alpha \left(t\right)\right)\;\left(a_{b}+\left(S_{a}\;M_{a}-I\right)\;f_{b}\left(t\right)\right)\;dt\;dt.$$

The solution for these equations is difficult to obtain in the general case for the whole trajectory, and a typical approach would be a numerical simulation.

### 2.2. Methodology and Considered Cases

However, the solution of Equations (6) and (7) can be found quite easily after assuming piecewise constant motion characteristics according to Equations (8)–(11):

(8) $$\begin{array}{cc} a(t) & =a=\mathrm{const}, \end{array}$$

(9) $$\begin{array}{cc} \omega (t) & =\omega =\mathrm{const}, \end{array}$$

(10) $$\begin{array}{cc} u(t) & =u_{0}+a\;t, \end{array}$$

(11) $$\begin{array}{cc} R(\alpha (t)) & =\exp\left(-\Omega \;t\right)\;R\left(\alpha_{0}\right), \end{array}$$

where $\Omega ={\left[\omega \right]}_{\times }$ is the angular rate skew-symmetric matrix, and exp(·) is a matrix exponent.

The symbolic solution is still a complicated task for a human, which is probably the rationale for why such an approach is not popular. However, the calculations are simple with the help of symbolic computing software. In the case of this paper, Matlab with symbolic math toolbox was used. Nevertheless, the results obtained still turned out to be very extensive. Thus, to facilitate the reader’s understanding of the results presented, the analysis does not consider the whole assumed trajectory, but decomposes it into individual stages, considering each manoeuvre separately and analysing changes in the obtained error equations. The cases considered are summarised in Table 1.

To maintain the details of the presented results and direct insight into the emerging dependencies, the individual components of the errors were expanded. However, for this to be possible, a detailed model of the measurement system had to be adopted by specifying the exact structure of the sensor model matrices.

#### Detailed Measurement System Model

Based on the review of commercially available sensors’ documentation, it is possible to distinguish the parameters that are given in almost every specification, so their use in the model will allow a direct comparison of IMU capabilities by substituting the parameters of their sensors into the formulas obtained from the analysis. The parameters thus selected are summarised in Table 2.

It should be noted that the listed parameters are typical for IMUs available on the market [1], which represent only a cross section of existing sensor technologies [17]. Therefore, it should be assumed that the models presented below based on these parameters are applicable to optical gyroscopes, i.e., ring laser gyroscope (RLG) or fibre optic gyroscope (FOG) and sensors manufactured with micro-electro-mechanical systems (MEMS) technology.

Triads of sensors are used in the considered measuring system, while in the specification, only one value of each parameter is given, and identical errors of each sensor in the triad were assumed. It was also assumed that gyroscopes and accelerometers have the same error matrix structures. The adopted matrices are shown in Equations (12)–(14):

(12) $$\begin{array}{cc} \;S_{i} & =\left[\begin{array}{ccc} 1+\epsilon_{i} & 0 & 0\\ 0 & 1+\epsilon_{i} & 0\\ 0 & 0 & 1+\epsilon_{i} \end{array}\right], \end{array}$$

(13) $$\begin{array}{cc} \;M_{i} & =\left[\begin{array}{ccc} \sqrt{1-\delta_{i}^{2}} & \delta_{i}/\sqrt{2} & \delta_{i}/\sqrt{2}\\ \delta_{i}/\sqrt{2} & \sqrt{1-\delta_{i}^{2}} & \delta_{i}/\sqrt{2}\\ \delta_{i}/\sqrt{2} & \delta_{i}/\sqrt{2} & \sqrt{1-\delta_{i}^{2}} \end{array}\right], \end{array}$$

(14) $$\begin{array}{cc} \;\Omega_{g} & =\left[\begin{array}{ccc} \omega_{g} & 0 & 0\\ 0 & \omega_{g} & 0\\ 0 & 0 & \omega_{g} \end{array}\right], \end{array}$$

where index *i* should be replaced by *a* for accelerometers or ω for gyroscopes, while $\delta_{i}=\sin\left(\alpha_{i}\right)$, and $\alpha_{i}$ is sensor input axis misalignment [18].

### 2.3. Notes on Analysis

In the analysis of the cases where rotation occurs, sensor measurement errors due to measurement noise have been omitted. The analysis of the impact of stochastic errors in a rotating navigation system is addressed, e.g., in [19].

It is assumed that the object makes *coordinated* turns. That is, an object moving at speed u≠0 cannot change orientation without changing the direction of motion (except in the case of rotation along the direction of motion), and the resulting centripetal acceleration is equal to ω×u. This assumption follows from the practical consideration that most objects move in a particular direction—*forward*. The exception is the case of free fall, for which motion is considered with velocity u≈0 or in orbit, where the constraint may not hold.

In the analysis, the error of the initial velocity is expressed in the navigation coordinate system. However, if the initial velocity in the coordinate system is determined by the velocity expressed in the body system and the orientation of the body, the initial velocity error results from the velocity errors in the body system and the orientation error, as in Equation (15):

(15) $$v_{e,0}=\alpha_{e,0}\times v_{0}+R^{T}\left(\alpha_{0}\right)\;u_{e,0}.$$

## 3. Results

The analysis of the obtained results begins with the simplest case, and we gradually move through the more complicated ones, successively adding the individual motion parameters, according to Table 1. We begin by analysing the error of orientation determination, as the error obtained will be further used to determine the position error.

The first two cases are the free fall, with the body coordinate system either aligned to the navigation coordinate system or in an arbitrary orientation. As can be seen in Equations (16) and (17), orientation error for these cases consists of only initial and time-dependent errors (bias and random walk).

(16) $$\alpha_{e}\left(t\right)=\left[\begin{array}{c} \alpha_{e,0,x}\\ \alpha_{e,0,y}\\ \alpha_{e,0,z} \end{array}\right]+\left[\begin{array}{c} \omega_{b,x}\;t+\mathrm{arw}\;\sqrt{t}\\ \omega_{b,y}\;t+\mathrm{arw}\;\sqrt{t}\\ \omega_{b,z}\;t+\mathrm{arw}\;\sqrt{t} \end{array}\right]$$

(17) $$\alpha_{e}\left(t\right)=\left[\begin{array}{c} \alpha_{e,0,x}\\ \alpha_{e,0,y}\\ \alpha_{e,0,z} \end{array}\right]+R^{T}\left(\alpha_{0}\right)\;\left[\begin{array}{c} \omega_{b,x}\;t+\mathrm{arw}\;\sqrt{t}\\ \omega_{b,y}\;t+\mathrm{arw}\;\sqrt{t}\\ \omega_{b,z}\;t+\mathrm{arw}\;\sqrt{t} \end{array}\right]$$

If we place the system stationary, as in Case 3 shown in Equation (18), an error appears due to the acceleration sensitivity of the gyroscopes. Generalising to an arbitrary orientation in Case 4, presented in Equation (19), the only change is a consequent error rotation. Due to the change in the basis of the g-sensitivity matrix and the appearance of multiple trigonometric functions, the gravitational acceleration-dependent term is not expanded and is written in general matrix form.

(18) $$\alpha_{e}\left(t\right)=\left[\begin{array}{c} \alpha_{e,0,x}\\ \alpha_{e,0,y}\\ \alpha_{e,0,z} \end{array}\right]+\left[\begin{array}{c} \omega_{b,x}\;t+\mathrm{arw}\;\sqrt{t}\\ \omega_{b,y}\;t+\mathrm{arw}\;\sqrt{t}\\ \omega_{b,z}\;t+\mathrm{arw}\;\sqrt{t} \end{array}\right]+\omega_{g}\;t\;\left[\begin{array}{c} -\frac{1}{\sqrt{2}}\;\delta_{w}\\ -\frac{1}{\sqrt{2}}\;\delta_{w}\\ -\sqrt{1-{\delta_{w}}^{2}} \end{array}\right]\;g_{0}$$

(19) $$\alpha_{e}\left(t\right)=\left[\begin{array}{c} \alpha_{e,0,x}\\ \alpha_{e,0,y}\\ \alpha_{e,0,z} \end{array}\right]+R^{T}\left(\alpha_{0}\right)\;\left[\begin{array}{c} \omega_{b,x}\;t+\mathrm{arw}\;\sqrt{t}\\ \omega_{b,y}\;t+\mathrm{arw}\;\sqrt{t}\\ \omega_{b,z}\;t+\mathrm{arw}\;\sqrt{t} \end{array}\right]+t\;R^{T}\left(\alpha_{0}\right)\;\Omega_{g}\;M_{\omega }\;R\left(\alpha_{0}\right)\;\left(-g\right)$$

In the case shown, the acceleration is the ground reaction against the gravitational force, but an identical term will appear for any acceleration a(t). It is interesting to note, however, that identical results to those presented by Equation (19) will also be obtained for Case 5, that is, when the object is moving in steady rectilinear motion in an arbitrary direction with arbitrary speed and orientation. It means that from the point of view of the orientation error does not matter whether the body is at rest or moves with constant velocity. Hence, in the case of steady motion, an analysis that only takes into account time-dependent errors is sufficient.

The next cases present formulas for the error of orientation determination for motion containing rotations. Case 6, shown in Equation (20), represents free fall, similar to that shown in Equation (16) but with rotation around the Z axis. Comparing the two equations, one can see that the errors depend on time (only bias in this case), as well as a gyroscope misalignment and scale factor errors. Most significantly, however, the errors in axes perpendicular to the rotation vector no longer increase linearly with time but have a limited maximum value. This implies a significant reduction in attitude errors and analytically demonstrates the correctness of results from the publications [4,7].

(20) $$\begin{array}{cc} \alpha_{e}\left(t\right)\; & =\left[\begin{array}{c} \alpha_{e,0,x}\\ \alpha_{e,0,y}\\ \alpha_{e,0,z} \end{array}\right]+\left[\begin{array}{c} \left(\frac{\omega_{b,x}}{w_{z}}+\frac{1}{\sqrt{2}}\;\delta_{w}\;\left(\epsilon_{w}+1\right)\right)\;\sin\left(t\;w_{z}\right)+\left(-\frac{2\;\omega_{b,y}}{w_{z}}-\sqrt{2}\;\delta_{w}\;\left(\epsilon_{w}+1\right)\right)\;{\sin\left(\frac{t\;w_{z}}{2}\right)}^{2}\\ \left(\frac{\omega_{b,y}}{w_{z}}+\frac{1}{\sqrt{2}}\;\delta_{w}\;\left(\epsilon_{w}+1\right)\right)\;\sin\left(t\;w_{z}\right)+\left(\frac{2\;\omega_{b,x}}{w_{z}}+\sqrt{2}\;\delta_{w}\;\left(\epsilon_{w}+1\right)\right)\;{\sin\left(\frac{t\;w_{z}}{2}\right)}^{2}\\ \left(\omega_{b,z}-w_{z}\;\left(1-\left(\epsilon_{a}+1\right)\;\sqrt{1-{\delta_{a}}^{2}}\right)\right)\;t \end{array}\right]\\ \; & =\left[\begin{array}{c} \alpha_{e,0,x}\\ \alpha_{e,0,y}\\ \alpha_{e,0,z} \end{array}\right]+R^{T}\left(\alpha_{0}\right)\;\int_{0}^{t}\exp\left(\Omega \;t\right)\;dt\;\left(\omega_{b}+\left(S_{\omega }\;M_{\omega }-I\right)\;\omega \right) \end{array}$$

Moreover, the results obtained extend the results from the publications mentioned above since, as can be seen from the Formulas (21) and (22) for Cases 7 and 8, respectively, the identical effect as for the gyroscope bias also occurs for g–sensitive errors emerging from gravitational and centripetal acceleration during circular motion.

(21) $$\begin{array}{cc} \alpha_{e}\left(t\right)\; & =\left[\begin{array}{c} \alpha_{e,0,x}\\ \alpha_{e,0,y}\\ \alpha_{e,0,z} \end{array}\right]+\left[\begin{array}{c} \left(\frac{\omega_{b,x}}{w_{z}}+\frac{1}{\sqrt{2}}\;\delta_{w}\;\left(\epsilon_{w}+1\right)\right)\;\sin\left(t\;w_{z}\right)+\left(-\frac{2\;\omega_{b,y}}{w_{z}}-\sqrt{2}\;\delta_{w}\;\left(\epsilon_{w}+1\right)\right)\;{\sin\left(\frac{t\;w_{z}}{2}\right)}^{2}\\ \left(\frac{\omega_{b,y}}{w_{z}}+\frac{1}{\sqrt{2}}\;\delta_{w}\;\left(\epsilon_{w}+1\right)\right)\;\sin\left(t\;w_{z}\right)+\left(\frac{2\;\omega_{b,x}}{w_{z}}+\sqrt{2}\;\delta_{w}\;\left(\epsilon_{w}+1\right)\right)\;{\sin\left(\frac{t\;w_{z}}{2}\right)}^{2}\\ \left(\omega_{b,z}-w_{z}\;\left(1-\left(\epsilon_{a}+1\right)\;\sqrt{1-{\delta_{a}}^{2}}\right)\right)\;t \end{array}\right]\\ \; & +\omega_{g}\;\left[\begin{array}{c} -\frac{\sqrt{2}\;\delta_{w}\;\sin\left(t\;w_{z}\right)}{2\;w_{z}}+\frac{\sqrt{2}\;\delta_{w}\;{\sin\left(\frac{t\;w_{z}}{2}\right)}^{2}}{w_{z}}\\ -\frac{\sqrt{2}\;\delta_{w}\;\sin\left(t\;w_{z}\right)}{2\;w_{z}}-\frac{\sqrt{2}\;\delta_{w}\;{\sin\left(\frac{t\;w_{z}}{2}\right)}^{2}}{w_{z}}\\ -t\;\sqrt{1-{\delta_{w}}^{2}} \end{array}\right]\;g_{0}\\ \; & =\left[\begin{array}{c} \alpha_{e,0,x}\\ \alpha_{e,0,y}\\ \alpha_{e,0,z} \end{array}\right]+R^{T}\left(\alpha_{0}\right)\;\int_{0}^{t}\exp\left(\Omega \;t\right)\;dt\;\left(\omega_{b}+\left(S_{\omega }\;M_{\omega }-I\right)\;\omega \right)\\ \; & +R^{T}\left(\alpha_{0}\right)\;\int_{0}^{t}\exp\left(\Omega \;t\right)\;\Omega_{g}\;M_{\omega }\;\exp\left(-\Omega \;t\right)\;dt\;R\left(\alpha_{0}\right)\;\left(-g\right) \end{array}$$

(22) $$\begin{array}{cc} \alpha_{e}\left(t\right)\; & =\left[\begin{array}{c} \alpha_{e,0,x}\\ \alpha_{e,0,y}\\ \alpha_{e,0,z} \end{array}\right]+\left[\begin{array}{c} \left(\frac{\omega_{b,x}}{w_{z}}+\frac{1}{\sqrt{2}}\;\delta_{w}\;\left(\epsilon_{w}+1\right)\right)\;\sin\left(t\;w_{z}\right)+\left(-\frac{2\;\omega_{b,y}}{w_{z}}-\sqrt{2}\;\delta_{w}\;\left(\epsilon_{w}+1\right)\right)\;{\sin\left(\frac{t\;w_{z}}{2}\right)}^{2}\\ \left(\frac{\omega_{b,y}}{w_{z}}+\frac{1}{\sqrt{2}}\;\delta_{w}\;\left(\epsilon_{w}+1\right)\right)\;\sin\left(t\;w_{z}\right)+\left(\frac{2\;\omega_{b,x}}{w_{z}}+\sqrt{2}\;\delta_{w}\;\left(\epsilon_{w}+1\right)\right)\;{\sin\left(\frac{t\;w_{z}}{2}\right)}^{2}\\ \left(\omega_{b,z}-w_{z}\;\left(1-\left(\epsilon_{a}+1\right)\;\sqrt{1-{\delta_{a}}^{2}}\right)\right)\;t \end{array}\right]\\ \; & +\omega_{g}\;\left[\begin{array}{c} -\frac{\sqrt{2}\;\delta_{w}\;\sin\left(t\;w_{z}\right)}{2\;w_{z}}+\frac{\sqrt{2}\;\delta_{w}\;{\sin\left(\frac{t\;w_{z}}{2}\right)}^{2}}{w_{z}}\\ -\frac{\sqrt{2}\;\delta_{w}\;\sin\left(t\;w_{z}\right)}{2\;w_{z}}-\frac{\sqrt{2}\;\delta_{w}\;{\sin\left(\frac{t\;w_{z}}{2}\right)}^{2}}{w_{z}}\\ -\sqrt{1-{\delta_{w}}^{2}} \end{array}\right]\;g_{0}+\omega_{g}\;\left[\begin{array}{c} \frac{1}{\sqrt{2}}\;\delta_{w}\;\sin\left(t\;w_{z}\right)-2\;{\sin\left(\frac{t\;w_{z}}{2}\right)}^{2}\;\sqrt{1-{\delta_{w}}^{2}}\\ \sin\left(t\;w_{z}\right)\;\sqrt{1-{\delta_{w}}^{2}}+\sqrt{2}\;\delta_{w}\;{\sin\left(\frac{t\;w_{z}}{2}\right)}^{2}\\ +\frac{1}{\sqrt{2}}\;\delta_{w}\;t\;w_{z} \end{array}\right]\;u_{0,x}\\ \; & =\left[\begin{array}{c} \alpha_{e,0,x}\\ \alpha_{e,0,y}\\ \alpha_{e,0,z} \end{array}\right]+R^{T}\left(\alpha_{0}\right)\;\int_{0}^{t}\exp\left(\Omega \;t\right)\;dt\;\left(\omega_{b}+\left(S_{\omega }\;M_{\omega }-I\right)\;\omega \right)\\ \; & +R^{T}\left(\alpha_{0}\right)\;\int_{0}^{t}\exp\left(\Omega \;t\right)\;\Omega_{g}\;M_{\omega }\;\exp\left(-\Omega \;t\right)\;dt\;R\left(\alpha_{0}\right)\;\left(-g\right)+R^{T}\left(\alpha_{0}\right)\;\int_{0}^{t}\exp\left(\Omega \;t\right)\;dt\;\Omega_{g}\;M_{\omega }\;\left(\omega \times u_{0}\right) \end{array}$$

However, it is only the addition of constant acceleration, addressed in Case 9 with the resulting orientation error shown in Equation (23), that causes the orientation error to increase linearly over time.

(23) $$\begin{array}{cc} \alpha_{e}\left(t\right)\; & =\left[\begin{array}{c} \alpha_{e,0,x}\\ \alpha_{e,0,y}\\ \alpha_{e,0,z} \end{array}\right]+\left[\begin{array}{c} \left(\frac{\omega_{b,x}}{w_{z}}+\frac{1}{\sqrt{2}}\;\delta_{w}\;\left(\epsilon_{w}+1\right)\right)\;\sin\left(t\;w_{z}\right)+\left(-\frac{2\;\omega_{b,y}}{w_{z}}-\sqrt{2}\;\delta_{w}\;\left(\epsilon_{w}+1\right)\right)\;{\sin\left(\frac{t\;w_{z}}{2}\right)}^{2}\\ \left(\frac{\omega_{b,y}}{w_{z}}+\frac{1}{\sqrt{2}}\;\delta_{w}\;\left(\epsilon_{w}+1\right)\right)\;\sin\left(t\;w_{z}\right)+\left(\frac{2\;\omega_{b,x}}{w_{z}}+\sqrt{2}\;\delta_{w}\;\left(\epsilon_{w}+1\right)\right)\;{\sin\left(\frac{t\;w_{z}}{2}\right)}^{2}\\ \left(\omega_{b,z}-w_{z}\;\left(1-\left(\epsilon_{a}+1\right)\;\sqrt{1-{\delta_{a}}^{2}}\right)\right)\;t \end{array}\right]\\ \; & +\omega_{g}\;\left[\begin{array}{c} -\frac{\sqrt{2}\;\delta_{w}\;\sin\left(t\;w_{z}\right)}{2\;w_{z}}+\frac{\sqrt{2}\;\delta_{w}\;{\sin\left(\frac{t\;w_{z}}{2}\right)}^{2}}{w_{z}}\\ -\frac{\sqrt{2}\;\delta_{w}\;\sin\left(t\;w_{z}\right)}{2\;w_{z}}-\frac{\sqrt{2}\;\delta_{w}\;{\sin\left(\frac{t\;w_{z}}{2}\right)}^{2}}{w_{z}}\\ -t\;\sqrt{1-{\delta_{w}}^{2}} \end{array}\right]\;g_{0}+\omega_{g}\;\left[\begin{array}{c} \frac{1}{\sqrt{2}}\;\delta_{w}\;\sin\left(t\;w_{z}\right)-2\;{\sin\left(\frac{t\;w_{z}}{2}\right)}^{2}\;\sqrt{1-{\delta_{w}}^{2}}\\ \sin\left(t\;w_{z}\right)\;\sqrt{1-{\delta_{w}}^{2}}+\sqrt{2}\;\delta_{w}\;{\sin\left(\frac{t\;w_{z}}{2}\right)}^{2}\\ \frac{1}{\sqrt{2}}\;\delta_{w}\;w_{z}\;t \end{array}\right]\;u_{0,x}\\ \; & +\omega_{g}\;\left[\begin{array}{c} t\;\sqrt{1-{\delta_{w}}^{2}}-\frac{2\;\sqrt{2}\;\delta_{w}\;{\sin\left(\frac{t\;w_{z}}{2}\right)}^{2}}{w_{z}}\\ -\frac{1}{\sqrt{2}}\;\delta_{w}\;t+\frac{\sqrt{2}\;\delta_{w}\;\sin\left(t\;w_{z}\right)}{w_{z}}\\ \frac{1}{\sqrt{2}}\;\delta_{w}\;t \end{array}\right]\;a_{\mathrm{sx}}+\omega_{g}\;\left[\begin{array}{c} \frac{1}{\sqrt{2}}\;\delta_{w}\;t\;\sin\left(t\;w_{z}\right)-2\;t\;{\sin\left(\frac{t\;w_{z}}{2}\right)}^{2}\sqrt{1-{\delta_{w}}^{2}}\\ t\;\sin\left(t\;w_{z}\right)\;\sqrt{1-{\delta_{w}}^{2}}+\sqrt{2}\;\delta_{w}\;t\;{\sin\left(\frac{t\;w_{z}}{2}\right)}^{2}\\ \frac{1}{2\;\sqrt{2}}\;\delta_{w}\;w_{z}\;t^{2} \end{array}\right]\;a_{\mathrm{sx}}\\ \; & =\left[\begin{array}{c} \alpha_{e,0,x}\\ \alpha_{e,0,y}\\ \alpha_{e,0,z} \end{array}\right]+R^{T}\left(\alpha_{0}\right)\;\int_{0}^{t}\exp\left(\Omega \;t\right)\;dt\;\left(\omega_{b}+\left(S_{\omega }\;M_{\omega }-I\right)\;\omega \right)\\ \; & +R^{T}\left(\alpha_{0}\right)\;\int_{0}^{t}\exp\left(\Omega \;t\right)\;\Omega_{g}\;M_{\omega }\;\exp\left(-\Omega \;t\right)\;dt\;R\left(\alpha_{0}\right)\;\left(-g\right)\\ \; & +R^{T}\left(\alpha_{0}\right)\;\int_{0}^{t}\exp\left(\Omega \;t\right)\;t\;dt\;\Omega_{g}\;M_{\omega }\;\left(\omega \times a\right)+R^{T}\left(\alpha_{0}\right)\;\int_{0}^{t}\exp\left(\Omega \;t\right)\;dt\;\Omega_{g}\;M_{\omega }\;\left(\omega \times u_{0}+a\right) \end{array}$$

On the other hand, the rotation of the navigation system does not affect the orientation error in the axis parallel to the rotation vector. Instead, continuous rotation causes an increase in the error of orientation determination in this axis due to the linearly increasing scale factor error.

The presented formulas provide the results for the case when the velocity and acceleration are in the X axis of the body coordinate system, and the rotation is around the Z axis of the body coordinate system. However, the presented effects are satisfied for any cases of rotation. The observed effects will occur, just on a different plane. To cover other cases, more general matrix formulas are also presented for Equations (20)–(23).

In the following part, we again pass through all the considered manoeuvres, this time analysing the position determination error. Considering Cases 1 and 2 again, this time in terms of the position determination error shown in Equations (24) and (25), one can find a similar relationship as for the orientation errors from Equations (16) and (17)—the error depends only on the initial orientation errors and the time-dependent errors (bias and random walk). It can also be seen that as long as the specific force is equal to zero, the orientation and position errors are independent of each other (assuming the initial velocity error does not depend on the orientation errors).

(24) $$p_{e}\left(t\right)=\left[\begin{array}{c} p_{e,0,x}+t\;v_{e,0,x}\\ p_{e,0,y}+t\;v_{e,0,y}\\ p_{e,0,z}+t\;v_{e,0,z} \end{array}\right]+\left[\begin{array}{c} \frac{1}{2}\;a_{b,x}\;t^{2}+\frac{2}{3}\;\mathrm{vrw}\;t^{3/2}\\ \frac{1}{2}\;a_{b,y}\;t^{2}+\frac{2}{3}\;\mathrm{vrw}\;t^{3/2}\\ \frac{1}{2}\;a_{b,z}\;t^{2}+\frac{2}{3}\;\mathrm{vrw}\;t^{3/2} \end{array}\right]$$

(25) $$p_{e}\left(t\right)=\left[\begin{array}{c} p_{e,0,x}+t\;v_{e,0,x}\\ p_{e,0,y}+t\;v_{e,0,y}\\ p_{e,0,z}+t\;v_{e,0,z} \end{array}\right]+R^{T}\left(\alpha_{0}\right)\left[\begin{array}{c} \frac{1}{2}\;a_{b,x}\;t^{2}+\frac{2}{3}\;\mathrm{vrw}\;t^{3/2}\\ \frac{1}{2}\;a_{b,y}\;t^{2}+\frac{2}{3}\;\mathrm{vrw}\;t^{3/2}\\ \frac{1}{2}\;a_{b,z}\;t^{2}+\frac{2}{3}\;\mathrm{vrw}\;t^{3/2} \end{array}\right]$$

Only for Cases 3 and 4, shown in Equations (26) and (27), respectively, does the position determination error include the misalignment and scale factor errors of accelerometers and incorporate the effect of orientation error on the position determination error. Leaving aside the scale and misalignment errors of accelerometers, Equation (26) is typically used to determine the accuracy of a navigation system based on navigation duration. Hence, the influence of other sources addressed in the following equations is neglected.

(26) $$\begin{array}{cc} p_{e}\left(t\right)\; & =\left[\begin{array}{c} p_{e,0,x}+t\;v_{e,0,x}\\ p_{e,0,y}+t\;v_{e,0,y}\\ p_{e,0,z}+t\;v_{e,0,z} \end{array}\right]+\left[\begin{array}{c} \frac{1}{2}\;a_{b,x}\;t^{2}+\frac{2}{3}\;\mathrm{vrw}\;t^{3/2}\\ \frac{1}{2}\;a_{b,y}\;t^{2}+\frac{2}{3}\;\mathrm{vrw}\;t^{3/2}\\ \frac{1}{2}\;a_{b,z}\;t^{2}+\frac{2}{3}\;\mathrm{vrw}\;t^{3/2} \end{array}\right]+\frac{1}{2}\;t^{2}\;\left[\begin{array}{c} -\frac{1}{\sqrt{2}}\;\delta_{a}\;\left(\epsilon_{a}+1\right)\\ -\frac{1}{\sqrt{2}}\;\delta_{a}\;\left(\epsilon_{a}+1\right)\\ 1-\sqrt{1-{\delta_{a}}^{2}}\;\left(\epsilon_{a}+1\right) \end{array}\right]\;g_{0}\\ \; & +\left[\begin{array}{c} -\frac{1}{2}\;\alpha_{e,0,y}\;t^{2}-\frac{1}{6}\;\omega_{b,y}\;t^{3}-\frac{4}{15}\;\mathrm{arw}\;t^{5/2}+\frac{\sqrt{2}}{12}\;\delta_{w}\;\omega_{g}\;g_{0}\;t^{3}\\ \frac{1}{2}\;\alpha_{e,0,x}\;t^{2}+\frac{1}{6}\;\omega_{b,x}\;t^{3}+\frac{4}{15}\;\mathrm{arw}\;t^{5/2}-\frac{\sqrt{2}}{12}\;\delta_{w}\;\omega_{g}\;g_{0}\;t^{3}\\ 0 \end{array}\right]\;g_{0} \end{array}$$

Due to the increasing complexity of the relationships for Case 4 to maintain readability, some errors in Equation (27) are presented in a general matrix form. In the steady rectilinear motion considered in Case 5, similar to the orientation determination error, the position determination error does not depend on the direction and velocity of the object motion, and the errors equation is identical to Case 4, presented in Equation (27).

(27) $$\begin{array}{cc} p_{e}\left(t\right)\; & =\left[\begin{array}{c} p_{e,0,x}+t\;v_{e,0,x}\\ p_{e,0,y}+t\;v_{e,0,y}\\ p_{e,0,z}+t\;v_{e,0,z} \end{array}\right]+R^{T}\left(\alpha_{0}\right)\;\left[\begin{array}{c} \frac{1}{2}\;a_{b,x}\;t^{2}+\frac{2}{3}\;\mathrm{vrw}\;t^{3/2}\\ \frac{1}{2}\;a_{b,y}\;t^{2}+\frac{2}{3}\;\mathrm{vrw}\;t^{3/2}\\ \frac{1}{2}\;a_{b,z}\;t^{2}+\frac{2}{3}\;\mathrm{vrw}\;t^{3/2} \end{array}\right]+\frac{1}{2}\;t^{2}\;R^{T}\left(\alpha_{0}\right)\;\left(S_{a}\;M_{a}-I\right)\;R\left(\alpha_{0}\right)\;\left(-g\right)\\ \; & +\left(\frac{1}{2}\;t^{2}\;\left[\begin{array}{c} \alpha_{e,0,x}\\ \alpha_{e,0,y}\\ \alpha_{e,0,z} \end{array}\right]+R^{T}\left(\alpha_{0}\right)\;\left(\frac{1}{6}\;t^{3}\;\left[\begin{array}{c} \omega_{b,x}\\ \omega_{b,y}\\ \omega_{b,z} \end{array}\right]+\frac{4}{15}\;t^{5/2}\;\left[\begin{array}{c} \mathrm{arw}\\ \mathrm{arw}\\ \mathrm{arw} \end{array}\right]+\frac{1}{6}\;t^{3}\;\omega_{g}\;M_{\omega }\;R\left(\alpha_{0}\right)\;\left(-g\right)\right)\right)\times \left(-g\right)\\ \; & =\left[\begin{array}{c} p_{e,0,x}+t\;v_{e,0,x}\\ p_{e,0,y}+t\;v_{e,0,y}\\ p_{e,0,z}+t\;v_{e,0,z} \end{array}\right]+R^{T}\left(\alpha_{0}\right)\;\left[\begin{array}{c} \frac{1}{2}\;a_{b,x}\;t^{2}+\frac{2}{3}\;\mathrm{vrw}\;t^{3/2}\\ \frac{1}{2}\;a_{b,y}\;t^{2}+\frac{2}{3}\;\mathrm{vrw}\;t^{3/2}\\ \frac{1}{2}\;a_{b,z}\;t^{2}+\frac{2}{3}\;\mathrm{vrw}\;t^{3/2} \end{array}\right]+\frac{1}{2}\;t^{2}\;R^{T}\left(\alpha_{0}\right)\;\left(S_{a}\;M_{a}-I\right)\;R\left(\alpha_{0}\right)\;\left(-g\right)\\ \; & +\int_{0}^{T}\int_{0}^{T}\alpha_{e}\left(t\right)\times \left(-g\right)\;dt\;dt \end{array}$$

Comparing Case 6 from Equation (28) to Case 1 from Equation (24) and Case 7 from Equation (29) to Case 3 from Equation (26), we see that in the case of position determination error, rotation of the navigation system allows the error dependence on time to be reduced from a quadratic to a linear dependence. The position error resulting from the orientation error in the equation will be similar to that for (27). However, due to the more complicated formula for orientation error (from Equation (21)), this component will not be expanded.

(28) $$\begin{array}{cc} p_{e}\left(t\right)\; & =\left[\begin{array}{c} p_{e,0,x}+t\;v_{e,0,x}\\ p_{e,0,y}+t\;v_{e,0,y}\\ p_{e,0,z}+t\;v_{e,0,z} \end{array}\right]+\left[\begin{array}{c} -a_{b,y}\;\left(\frac{t}{w_{z}}-\frac{\sin\left(t\;w_{z}\right)}{{w_{z}}^{2}}\right)+2\;a_{b,x}\;\frac{{\sin\left(\frac{t\;w_{z}}{2}\right)}^{2}}{{w_{z}}^{2}}\\ a_{b,x}\;\left(\frac{t}{w_{z}}-\frac{\sin\left(t\;w_{z}\right)}{{w_{z}}^{2}}\right)+2\;a_{b,y}\;\frac{{\sin\left(\frac{t\;w_{z}}{2}\right)}^{2}}{{w_{z}}^{2}}\\ +\frac{1}{2}\;a_{b,z}\;t^{2} \end{array}\right]\\ \; & =\left[\begin{array}{c} p_{e,0,x}+t\;v_{e,0,x}\\ p_{e,0,y}+t\;v_{e,0,y}\\ p_{e,0,z}+t\;v_{e,0,z} \end{array}\right]+R^{T}\left(\alpha_{0}\right)\;\int_{0}^{t}\int_{0}^{t}\exp\left(\Omega \;t\right)\;dt\;dt\;a_{b} \end{array}$$

(29) $$\begin{array}{cc} p_{e}\left(t\right)\; & =\left[\begin{array}{c} p_{e,0,x}+t\;v_{e,0,x}\\ p_{e,0,y}+t\;v_{e,0,y}\\ p_{e,0,z}+t\;v_{e,0,z} \end{array}\right]+\left[\begin{array}{c} -a_{b,y}\;\left(\frac{t}{w_{z}}-\frac{\sin\left(t\;w_{z}\right)}{{w_{z}}^{2}}\right)+2\;a_{b,x}\;\frac{{\sin\left(\frac{t\;w_{z}}{2}\right)}^{2}}{{w_{z}}^{2}}\\ a_{b,x}\;\left(\frac{t}{w_{z}}-\frac{\sin\left(t\;w_{z}\right)}{{w_{z}}^{2}}\right)+2\;a_{b,y}\;\frac{{\sin\left(\frac{t\;w_{z}}{2}\right)}^{2}}{{w_{z}}^{2}}\\ \frac{1}{2}\;a_{b,z}\;t^{2} \end{array}\right]\\ \; & +\left[\begin{array}{c} \frac{1}{\sqrt{2}}\;\delta_{a}\;\left(\epsilon_{a}+1\right)\;\left(\frac{t}{w_{z}}-\frac{\sin\left(t\;w_{z}\right)}{w_{z}^{2}}\right)+-\sqrt{2}\;\delta_{a}\;\left(\epsilon_{a}+1\right)\;\frac{{\sin\left(\frac{t\;w_{z}}{2}\right)}^{2}}{{w_{z}}^{2}}\\ -\frac{1}{\sqrt{2}}\;\delta_{a}\;\left(\epsilon_{a}+1\right)\;\left(\frac{t}{w_{z}}-\frac{\sin\left(t\;w_{z}\right)}{w_{z}^{2}}\right)+-\sqrt{2}\;\delta_{a}\;\left(\epsilon_{a}+1\right)\;\frac{{\sin\left(\frac{t\;w_{z}}{2}\right)}^{2}}{{w_{z}}^{2}}\\ \frac{1}{2}\;\left(1-\left(\epsilon_{a}+1\right)\;\sqrt{1-{\delta_{a}}^{2}}\right)\;t^{2} \end{array}\right]\;g_{0}+\int_{0}^{t}\int_{0}^{t}\alpha_{e}\left(t\right)\times \left(-g\right)\;dt\;dt\\ \; & =\left[\begin{array}{c} p_{e,0,x}+t\;v_{e,0,x}\\ p_{e,0,y}+t\;v_{e,0,y}\\ p_{e,0,z}+t\;v_{e,0,z} \end{array}\right]+R^{T}\left(\alpha_{0}\right)\;\int_{0}^{t}\int_{0}^{t}\exp\left(\Omega \;t\right)\;dt\;dt\;a_{b}\\ \; & +R^{T}\left(\alpha_{0}\right)\;\int_{0}^{t}\int_{0}^{t}\exp\left(\Omega \;t\right)\;\left(S_{a}\;M_{a}-I\right)\;\exp\left(-\Omega \;t\right)\;dt\;dt\;R\left(\alpha_{0}\right)\;\left(-g\right)+\int_{0}^{t}\int_{0}^{t}\alpha_{e}\left(t\right)\times \left(-g\right)\;dt\;dt \end{array}$$

The result for Case 8 in Equation (30) relative to the case in Equation (29) adds a component resulting from centripetal acceleration in a circular motion. The last of the analysed cases, presented in Equation (31), turned out to be too complex to be presented in an expanded form in this paper. Hence, only the general matrix form is presented. However, it is the most general form, including all motion parameters and arranged so that the interpretation of the individual components is straightforward.

(30) $$\begin{array}{cc} p_{e}\left(t\right)\; & =\left[\begin{array}{c} p_{e,0,x}+t\;v_{e,0,x}\\ p_{e,0,y}+t\;v_{e,0,y}\\ p_{e,0,z}+t\;v_{e,0,z} \end{array}\right]+\left[\begin{array}{c} -a_{b,y}\;\left(\frac{t}{w_{z}}-\frac{\sin\left(t\;w_{z}\right)}{{w_{z}}^{2}}\right)+2\;a_{b,x}\;\frac{{\sin\left(\frac{t\;w_{z}}{2}\right)}^{2}}{{w_{z}}^{2}}\\ a_{b,x}\;\left(\frac{t}{w_{z}}-\frac{\sin\left(t\;w_{z}\right)}{{w_{z}}^{2}}\right)+2\;a_{b,y}\;\frac{{\sin\left(\frac{t\;w_{z}}{2}\right)}^{2}}{{w_{z}}^{2}}\\ \frac{1}{2}\;a_{b,z}\;t^{2} \end{array}\right]\\ \; & +\left[\begin{array}{c} \frac{1}{\sqrt{2}}\;\delta_{a}\;\left(\epsilon_{a}+1\right)\;\left(\frac{t}{w_{z}}-\frac{\sin\left(t\;w_{z}\right)}{w_{z}^{2}}\right)+-\sqrt{2}\;\delta_{a}\;\left(\epsilon_{a}+1\right)\;\frac{{\sin\left(\frac{t\;w_{z}}{2}\right)}^{2}}{{w_{z}}^{2}}\\ -\frac{1}{\sqrt{2}}\;\delta_{a}\;\left(\epsilon_{a}+1\right)\;\left(\frac{t}{w_{z}}-\frac{\sin\left(t\;w_{z}\right)}{w_{z}^{2}}\right)+-\sqrt{2}\;\delta_{a}\;\left(\epsilon_{a}+1\right)\;\frac{{\sin\left(\frac{t\;w_{z}}{2}\right)}^{2}}{{w_{z}}^{2}}\\ \frac{1}{2}\;\left(1-\left(\epsilon_{a}+1\right)\;\sqrt{1-{\delta_{a}}^{2}}\right)\;t^{2} \end{array}\right]\;g_{0}\\ \; & +\left[\begin{array}{c} \left(1-\left(\epsilon_{a}+1\right)\;\sqrt{1-{\delta_{a}}^{2}}\right)\;\left(t-\frac{\sin\left(t\;w_{z}\right)}{w_{z}}\right)+\sqrt{2}\;\delta_{a}\;\left(\epsilon_{a}+1\right)\;\frac{{\sin\left(\frac{t\;w_{z}}{2}\right)}^{2}}{w_{z}}\\ \frac{1}{\sqrt{2}}\;\delta_{a}\;\left(\epsilon_{a}+1\right)\;\left(t-\frac{\sin\left(t\;w_{z}\right)}{w_{z}}\right)-2\;\left(1-\;\left(\epsilon_{a}+1\right)\;\sqrt{1-{\delta_{a}}^{2}}\right)\;\frac{{\sin\left(\frac{t\;w_{z}}{2}\right)}^{2}}{w_{z}}\\ \frac{1}{2\;\sqrt{2}}\;\delta_{a}\;w_{z}\;\left(\epsilon_{a}+1\right)\;t^{2} \end{array}\right]\;u_{0,x}\\ \; & +\int_{0}^{t}\int_{0}^{t}\alpha_{e}\left(t\right)\times \left(R^{T}\left(\alpha_{0}\right)\;\exp\left(\Omega \;t\right)\;\left(\omega \times u_{0}\right)-g\right)\;dt\;dt \end{array}$$

(31) $$\begin{array}{cc} p_{e}\left(t\right)\; & =p_{e,0}+t\;v_{e,0}+R^{T}\left(\alpha_{0}\right)\;\int_{0}^{t}\int_{0}^{t}\exp\left(\Omega \;t\right)\;dt\;dt\;a_{b}\\ \; & +R^{T}\left(\alpha_{0}\right)\;\int_{0}^{t}\int_{0}^{t}\exp\left(\Omega \;t\right)\;\left(S_{a}\;M_{a}-I\right)\;\exp\left(-\Omega \;t\right)\;dt\;dt\;R\left(\alpha_{0}\right)\;\left(-g\right)\\ \; & +R^{T}\left(\alpha_{0}\right)\;\int_{0}^{t}\int_{0}^{t}\exp\left(\Omega \;t\right)\;dt\;dt\;\left(S_{a}\;M_{a}-I\right)\;\left(a+\omega \times u_{0}\right)\\ \; & +R^{T}\left(\alpha_{0}\right)\;\int_{0}^{t}\int_{0}^{t}\exp\left(\Omega \;t\right)\;t\;dt\;dt\;\left(S_{a}\;M_{a}-I\right)\;\left(\omega \times a\right)\\ \; & +\int_{0}^{t}\int_{0}^{t}\alpha_{e}\left(t\right)\times \left(R^{T}\left(\alpha_{0}\right)\;\exp\left(\Omega \;t\right)\;\left(a+\omega \times u_{0}+\omega \times a\;t\right)-g\right)\;dt\;dt \end{array}$$

### Application of Proposed Method

An example of a practical application will be presented by analysing the accuracy of a navigation system for two cases: (1) a rotating stationary IMU, and (2) an IMU mounted on a rocket launched from a stationary ground launcher. In both cases, the results obtained will be compared with the common approach, which considers only time-dependent errors. The analysis considers the measurement system errors listed in Table 3 with values taken from the documentation of a selected tactical grade IMU available on the market [1].

The formulas derived earlier in the article were used to determine the covariance matrix of the navigation variables, i.e., $P\left(t\right)=E\left\{p_{e}\left(t\right)\;p_{e}{\left(t\right)}^{T}\right\}$, assuming that each parameter is random constant with normal distribution of zero mean and standard deviation specified in Table 3. In addition to the sensor parameters, initial position and velocity were assumed to be known exactly. Initial attitude and its errors were derived from inertial alignment, while 0.3 ${}^{\circ }$ has been assumed for the heading error.

**Figure 3:**
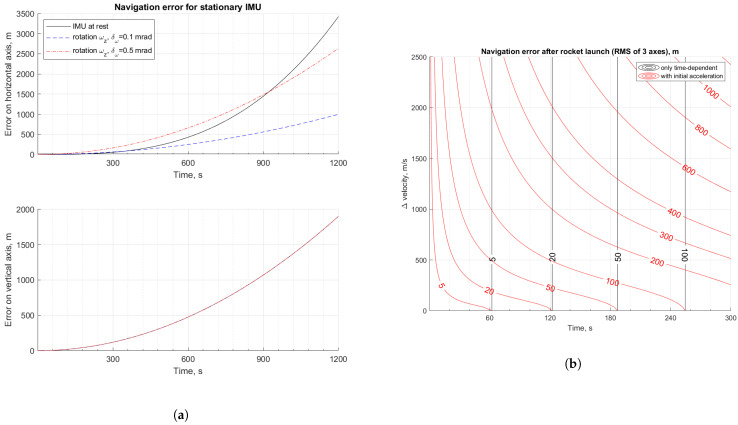
Influence of continuous rotation and launch acceleration on navigation system accuracy: (**a**) effect of stationary IMU rotation $\omega_{z}$ around vertical axis on the navigation system error for two values of gyroscopes misalignment $\delta_{\omega }$; errors along horizontal and vertical axes, respectively, in top and bottom plot; (**b**) contour plot showing effect of constant acceleration during launch on rocket navigation system accuracy, depending on target flight velocity ΔV and duration of the launch phase.

In Figure 3a, the black line shows the navigation error for a stationary IMU. The top plot shows the error for a single horizontal axis, while the bottom plot shows the error on a vertical axis. Dashed lines show the error for a stationary IMU but rotating around a vertical axis with rate $\omega_{z}=0.1$ rad/s. The Blue line corresponds to the parameters from Table 3, while the red line considers the case with worse gyroscope misalignment, i.e., $\delta_{\omega }=0.5$ mrad.

As can be seen for the blue line, the rotation of the measurement unit may improve navigation accuracy. However, as the IMU rotates continuously, there are additional errors due to the scale factor and misalignment errors. For high misalignment, continuous rotation improves accuracy in the long-term, but in a shorter term, accuracy may be even worse. What is more, continuous rotation around a vertical axis does not provide any improvement for navigation accuracy along that axis.

Figure 3b shows the contour plot of navigational errors for a rocket launched from a stationary ground launcher with 52${}^{\circ }$ elevation as a function of target flight velocity at motor burnout, ΔV, and duration of the launch phase. In the case considered, the rocket moves with constant acceleration along its longitudinal axis. Black lines are the contour plot for the case when only the duration of navigation is considered, i.e., according to Equation (27). These are vertical lines as the navigation error does not depend on target velocity. Red lines are the contour plot for analysis, taking into account accelerations during launch and using Equation (31). The values over the contour lines are the position error calculated as the RMS of errors on three orthogonal axes. As can be seen, acceleration results in a decrease of navigation accuracy due to motion-dependent errors, which is evident through the use of the derived equations.

The advantage of using the analytical form of the equations is the possibility to obtain the graphs shown in Figure 3 in one step by substituting the values of known parameters into the equations. In the case of simulation analysis, several simulations would have to be carried out for a range of parameter values.

## 4. Discussion

The most general form of the resulting equations is very complex, and direct analysis could be challenging. However, by analysing the individual cases from the simplest to the most complex, it was possible to gradually assemble the general expression of the complex model. Presenting the results in analytical form, particularly expanded and neatly arranged, gives a much better insight into the relationships than graphs do. Thus, dependencies can be made visible and understood.

The analysis shows that each fragment of the trajectory can be analysed separately, assuming piecewise motion parameter values. It is only necessary to assign consistent initial conditions resulting from the preceding fragments. Furthermore, the effect of each motion parameter within a single trajectory fragment can be analysed separately.

From the results presented, it can be seen that in the case of a free fall, the errors are only caused by bias and random walk. They therefore depend only on the duration of the navigation. It is worth noting that the same conclusion is presented in [10] for the simulation results. Furthermore, as can be seen from Equations (19) and (27), even for a moving object, as long as there are no accelerations, the orientation error does not affect the position error. These results indicate which parameters one should consider when selecting an inertial measurement system for a satellite, e.g., for an attitude and orbit control system (AOCS).

After taking gravity into account, the formulas obtained indicate that the errors accumulating during steady motion are not different from those accumulating for the stationary object and depend only on time. This observation leads to the conclusion that it is sufficient to determine the errors based on the navigation duration, complemented by a more detailed analysis only for fragments with high motion dynamics (significant accelerations or changes of direction—especially rapid ones). Thus, the analysis supports and extends the statement from [2] that for long-duration navigation with low dynamics, the most important source of navigation errors is the measurement error of the gyroscope.

The analysed cases of manoeuvres without rotation seem to be trivial. However, they made it possible to show the influence of the individual motion parameters and the parameters of the measurement system model on the resulting orientation or position determination errors. The analysis of manoeuvres with rotation gives more interesting results.

It can be seen that in many of the analysed cases, the orientation or position error is the product of the appropriate integral of exp(Ω(t)) and a constant vector. Equation (32) presents the first integral of exp(Ω(t)) for rotation around the Z axis.

(32) $$\int_{0}^{t}\exp\left(\Omega \;t\right)\;dt=\left[\begin{array}{ccc} \frac{\sin\left(t\;w_{z}\right)}{w_{z}}\; & -\frac{2\;{\sin\left(\frac{t\;w_{z}}{2}\right)}^{2}}{w_{z}}\; & 0\\ \frac{2\;{\sin\left(\frac{t\;w_{z}}{2}\right)}^{2}}{w_{z}}\; & \frac{\sin\left(t\;w_{z}\right)}{w_{z}}\; & 0\\ 0 & 0 & t \end{array}\right]$$

It can already be seen that continuous rotation makes it possible to reduce the dependence of the error on time in the plane perpendicular to the axis of rotation by reducing the exponent on the variable *t* by 1. In the example shown, the maximum error is limited, and its amplitude depends on the rate of rotation. For $\omega_{z}\to 0$, the amplitude of the errors increases to infinity, but the period also increases. Effectively, the limit of the diagonal elements tends to *t*, while the off-diagonal tends to 0, which is expected.

However, the analysis so far assumes that the rotation rate as well as the other motion parameters are constant on the analysed fragment. As shown in [8], when the rotation rate changes, the experimental results can deviate from those determined analytically for the ideal case. It can also be seen from Equation (32) that if the rotation is not continuous, the largest errors occur for a single 90° turn. In addition, ref. [6] shows that in practice the increase in error can be caused by the irregularity of the rotation and wobbling of the turntable. Taking these cases into account would require further development of the proposed method.

It is considered that the proposed method should also allow an analysis of changing motion parameters. In the case of changing the rotational axis, a series of short consecutive fragments could be analysed, each with a slightly different axis of rotation. However, it may not be easy to present the results understandably. It could be a direction for further analysis.

Another noticeable problem is that the navigation error is only limited to directions perpendicular to the axis of rotation. In the parallel direction, the error can be even higher due to the scale factor error of the gyroscope. Analyses described in the literature and this paper concern rotation around a single axis. To ensure error reduction in each navigation axis, the rotation would have to be realised by a more complex motion in space.

As [2] notes, the effect of the orientation error is an incorrect projection of measured accelerations into the navigation coordinate system, with the largest position error occurring in the plane perpendicular to the resultant acceleration. Therefore, in the stationary case, the error is mainly in the horizontal plane, and the use of rotation about the vertical axis will provide the greatest benefit. When the object is moving, a rotation around an axis that follows the resultant acceleration vector may be an attractive approach. However, changes in the orientation of this vector must be reasonably smooth.

## 5. Conclusions

This paper presents the results of orientation and position error calculations for an unaided inertial navigation system under various manoeuvres. Contrary to most of the literature, an analytical method for the analysis of navigation errors has been proposed. The method used allowed the determination of general analytical formulas. Expanding the formulas obtained, it was shown that the analytical approach allows simple insight and understanding of the relationships involved. Therefore, authors consider it complementary to existing simulation analyses. The results obtained are consistent with the conclusions of existing studies. When analysing the consequences of setting the navigation system in motion, the exact model of the measurement system and the influence of motion-dependent errors were also considered. In this respect, the paper extends the analyses available in the literature. Based on the results obtained and those described in previous works, several conclusions are drawn regarding the propagation of navigation errors during either low- or high-motion dynamics. The method presented was originally developed for the analysis of attainable accuracy and selection of an inertial measurement system for a designed navigation system. Hence, the results obtained may allow a more accurate analysis of navigation systems and a more precise definition of the requirements for measurement systems. However, it was not intended for implementation on the target device and online operation, in which case its use may not be beneficial. A further development of the proposed method may concern the determination of accurate navigation error formulas that take into account non-constant motion parameters, e.g., a variable axis of rotation. However, from what has been conducted so far, it appears that the problem may not be the determination of the equations but their interpretation, as the formulas become extensive very quickly.

## Figures and Tables

**Table 1:** List of considered manoeuvres.

No. Case	$\alpha_{0}$	ω	$v_{0}$	*a*	Equation $\alpha_{e}$	Equation $p_{e}$	Motion Description
1	0	0	0	g	(16)	(24)	free fall
2	$\alpha_{0}$	0	0	g	(17)	(25)	free fall in arbitrary orientation
3	0	0	0	0	(18)	(26)	rest
4	$\alpha_{0}$	0	0	0	(19)	(27)	rest in arbitrary orientation
5	$\alpha_{0}$	0	$v_{0}$	0	(19)	(27)	steady rectilinear motion
6	0	$\left[\begin{array}{c} 0\\ 0\\ w_{z} \end{array}\right]$	0	g, $\dot{u}=0$	(20)	(28)	free fall with rotation
7	0	$\left[\begin{array}{c} 0\\ 0\\ w_{z} \end{array}\right]$	0	0	(21)	(29)	rotation without translation
8	0	$\left[\begin{array}{c} 0\\ 0\\ w_{z} \end{array}\right]$	$\left[\begin{array}{c} v_{0,x}\\ 0\\ 0 \end{array}\right]$	0	(22)	(30)	circular motion
9	0	$\left[\begin{array}{c} 0\\ 0\\ w_{z} \end{array}\right]$	$\left[\begin{array}{c} v_{0,x}\\ 0\\ 0 \end{array}\right]$	$\left[\begin{array}{c} a_{sx}\\ 0\\ 0 \end{array}\right]$	(23)	(31)	spiral motion

**Table 2:** The most common parameters used to describe the IMU (based on the review of the existing IMUs’ documentation).

Symbol	Parameter	Units
Gyroscopes		
—	Input range	${}^{\circ }/s$
$\omega_{b}$	Bias instability	${}^{\circ }/s$ or ${}^{\circ }/h$
$\epsilon_{\omega }$	Scale factor accuracy	% or ppm
ARW	Angle Random Walk	${}^{\circ }/\sqrt{h}$
—	Noise density	${(}^{\circ }/s)/\sqrt{\mathrm{Hz}}$
$\alpha_{\omega }$	Input axis misalignment	${}^{\circ }$ or mrad
—	Bandwidth	Hz
$\omega_{g}$	Bias acceleration sensitivity	${(}^{\circ }/s)/g$
Accelerometers		
—	Input range	g
$a_{b}$	Bias instability	mg
$\epsilon_{a}$	Scale factor accuracy	% or ppm
VRW	Velocity Random Walk	$\left(m/s\right)/\sqrt{h}$
—	Noise density	$\mathrm{mg}/\sqrt{\mathrm{Hz}}$
$\alpha_{a}$	Input axis misalignment	${}^{\circ }$ or mrad
—	Bandwidth	Hz

**Table 3:** Parameters of sensors used in analysis.

Parameter	Value (1σ)	Units
Gyroscopes		
bias	0.25	${}^{\circ }/h$
scale factor error	100	ppm
misalignment	0.1	mrad
*g*-sensitivity	0.001	${(}^{\circ }/s)/g$
Accelerometers		
bias	0.1	mg
scale factor error	250	ppm
misalignment	0.1	mrad

## Data Availability

Not applicable.

## Abbreviations

The following abbreviations are used in this manuscript:

AOCS	Attitude and Orbit Control System
ARW	Angle Random Walk
FOG	Fibre Optic Gyroscope
IMU	Inertial Measurement Unit
INS	Inertial Navigation System
MEMS	Micro-Electro-Mechanical Systems
RLG	Ring Laser Gyroscope
VRW	Velocity Random Walk
